# Intermediate dose enoxaparin in hospitalized patients with moderate-severe COVID-19: a pilot phase II single-arm study, INHIXACOVID19

**DOI:** 10.1186/s12879-023-08297-7

**Published:** 2023-10-24

**Authors:** B. Cosmi, M. Giannella, G. Fornaro, F. Cristini, A. Patacca, A. Castagna, F. Mazzaferri, S. Testa, A. Pan, M. Lupi, P. Brambilla, A. Montineri, S. Frattima, E. G. Bignami, M. Salvetti, G. De Stefano, E. Grandone, G. Di Perri, R. Rozzini, A. Stella, A. Romagnoli, F. Drago, P. Viale

**Affiliations:** 1 Angiology and Blood Coagulation Unit, IRCCS Azienda Ospedaliero-Universitaria di Bologna, Via Albertoni, 15, Bologna, Italy; 2 Angiology and Blood Coagulation Unit, Department of Medical and Surgical Sciences, University of Bologna, Bologna, Italy; 3 Infectious Diseases Unit, Department of Medical and Surgical Sciences, Policlinico Sant’Orsola IRCSS, University of Bologna, Via Massarenti 11, Bologna, 40138 Italy; 4 Infectious Disease Unit, Forlì and Cesena Hospiitals, Forlì-Cesena, Italy; 5 Clinica di Malattie Infettive, Università Vita-Salute, IRCCS San Raffaele Hospital, Milan, Italy; 6 Division of Infectious Diseases, Department of Medicine, Verona University Hospital, Verona, Italy; 7 Haemostasis and Thrombosis Center, ASST Cremona, Cremona, Italy; 8 Infectious Disease Unit, ASST Cremona, Cremona, Italy; 9 San Marco Hospital, Catania, Italy; 10 Carlo Poma Hospital, Mantua, Italy; 11 Anesthesiology, Critical Care and Pain Medicine Division, Department of Medicine and Surgery, University of Parma, Parma, Italy; 12 ASST Spedali Civili di Brescia and University of Brescia, Brescia, Italy; 13 San Carlo Hospital Potenza, Potenza, Italy; 14 Fondazione “Casa Sollievo della Sofferenza” San Giovanni Rotondo, Department Medical and Surgical Sciences, University of Foggia, Foggia, Italy; 15 Ob/Gyn First Sechenov University, Moscow, Russia; 16 Amedeo di Savoia Hospital, Turin, Italy; 17 Dipartimento di Geraitria, Unità di cura subintensiva- Unità di Geriatria per Acuti, Unità di attività subacute,Poliambulanza Hospital, Brescia, Italy; 18 Department of Speciality Diagnostics and Experimental Medicine (DIMES), Sant’Orsola Hospital University of Bologna, Bologna, Italy; 19 Ricerche Nuove, Pisa, Italy; 20 University of Catania (UNICT), Catania, Italy

**Keywords:** COVID-19, Enoxaparin, Venous thromboembolism, Thromboprofilaxis, Major bleeding

## Abstract

**Background:**

Randomized clinical trials in non-critically ill COVID-19 patients showed that therapeutic-dose heparin increased survival with reduced organ support as compared with usual-care thromboprophylaxis, albeit with increased bleeding risk. The purpose of the study is to assess the safety of intermediate dose enoxaparin in hospitalized patients with moderate to severe COVID-19.

**Methods:**

A phase II single-arm interventional prospective study including patients receiving intermediate dose enoxaparin once daily according to body weight: 60 mg for 45–60 kg, 80 mg for 61–100 kg or 100 mg for > 100 kg for 14 days, with dose adjustment according to anti-factor Xa activity (target range: 0.4–0.6 UI/ml); an observational cohort (OC) included patients receiving enoxaparin 40 mg day for comparison. Follow-up was 90 days. Primary outcome was major bleeding within 30 and 90 days after treatment onset. Secondary outcome was the composite of all-cause 30 and 90-day mortality rates, disease severity at the end of treatment, intensive care unit (ICU) admission and length of ICU stay, length of hospitalization. All outcomes were adjudicated by an independent committee and analyzed before and after propensity score matching (PSm).

**Results:**

Major bleeding was similar in IC (1/98 1.02%) and in the OC (none), with only one event observed in a patient receiving concomitantly anti-platelet therapy. The composite outcome was observed in 53/98 patients (54%) in the IC and 132/203 (65%) patients in the OC (*p* = 0.07) before PSm, while it was observed in 50/90 patients (55.6%) in the IC and in 56/90 patients (62.2%) in the OC after PSm (*p* = 0.45). Length of hospitalization was lower in the IC than in OC [median 13 (IQR 8–16) vs 14 (11–21) days, *p* = 0.001], however it lost statistical significance after PSm (*p* = 0.08). At 30 days, two patients had venous thrombosis and two pulmonary embolism in the OC. Time to first negative RT-PCR were similar in the two groups.

**Conclusions:**

Weight adjusted intermediate dose heparin with anti-FXa monitoring is safe with potential positive impact on clinical course in COVID-19 non-critically ill patients.

**Trial registration:**

The study INHIXACOVID19 was registred on ClinicalTrials.gov with the trial registration number (TRN) NCT04427098 on 11/06/2020.

**Supplementary Information:**

The online version contains supplementary material available at 10.1186/s12879-023-08297-7.

## Background

Governments and health official worldwide are still facing the challenge of the severe respiratory syndrome epidemic due to the Coronavirus Disease 19 (COVID-19) [1] which was declared as a pandemic disease by WHO at the beginning of 2020 [2]. The disease encompasses four stages depending on clinical severity: mild, moderate, severe and critical and it can rapidly progress respiratory failure requiring hospital admission for respiratory support up to invasive ventilation [1, 3]. Hospitalized patients are inherently at high risk of venous thromboembolism (VTE) as acute infections are strong prothrombotic stimuli [4, 5]. An overall prevalence of VTE among patients with COVID-19 of 14.1% was found in a meta-analysis of 66 studies, with the highest incidence (22.7%) among those admitted to intensive care units (ICUs) [6]. In these patients routine pharmacological thromboprophylaxis has been recommended by WHO since the beginning of the pandemic [7]. In addition, COVID-19 patients exhibit an enhanced systemic hypercoagulable state as SARS-CoV-2 leads to diffuse endothelial damage [8]. SARS-COV-2 binds to the ACE2 receptor which normally degrades angiotensin II, and SARS-CoV-2–mediated downregulation of ACE2 leads to accumulation of angiotensin II, which may contribute to a procoagulant state [9, 10]. The endothelial damage and the inflammatory host response can be characterized by excessive immune activation and cytokine storm, which promotes hypercoagulability with both macrovascular and microvascular thrombotic complications [11, 12]. A pathology hallmark of COVID-19 infection is diffuse small vessel (venule, arteriole, and capillary) platelet–fibrin thrombosis and intravascular megakaryocytes in all major organs, including the heart, lungs, kidneys, liver and mesenteric fat [9, 10]. As a result, standard pharmacological prophylaxis with low molecular weight heparin (LMWH) or unfractionated heparin (UFH) may be insufficient in these patients as failure rates are not negligible (5–15% but 20–41% if arterial events are considered), especially in patients admitted to ICU who are characterized by a dynamic day-to-day variation of both thromboembolic and bleeding risk [13, 14]. These data have prompted the search for a better approach to pharmacological thromboprophylaxis, especially regarding its intensity. At least 75 trials of different antithrombotic strategies for COVID-19 have been registered, the majority of which employ heparin either unfractionated or LMWH [15]. Parenteral drugs such as heparins either unfractionated or LMWH may be preferred in acutely ill patients, also given drug-drug interaction with direct oral anticoagulants and some anti-viral regimens [16]. Several studies have evaluated the efficacy and safety escalated doses of heparin when compared to standard thromboprophylactic doses in hospitalized COVID-19 patients. These studies have also considered heparin role on the progression of disease measured as organ support free days, due to its potential ability to inhibit pulmonary micro-thrombosis and also its antiviral and anti-inflammatory properties [17].

Studies in ICU patients, such as the INSPIRATION trial, showed no benefit of intermediate doses of LMWH when compared with standard-dose prophylaxis, on the primary outcome (a composite of adjudicated acute VTE, arterial thrombosis, treatment with extracorporeal membrane oxygenation, or death) with more bleeding in the intermediate-dose group [18]. The international, multiplatform, randomized clinical trials, combining data of three studies ACTIV-4a REMAP-CAP and ATTACC, compared standard with therapeutic heparin thromboprophylaxis in COVID-19 patients. The trial in critically ill patients was stopped after enrolling 1098 patients when the prespecified criterion for futility was met for therapeutic-dose anticoagulation. The latter did not improve the primary outcome of days without organ support and was associated with more major bleeding complications than standard prophylaxis (3.8 vs. 2.3%) [19]. On the contrary, in 2219 noncritically ill patients with COVID-19, therapeutic-dose anticoagulation with heparin increased the probability of survival to hospital discharge with reduced use of cardiovascular or respiratory organ support as compared with usual-care thromboprophylaxis, albeit with increased risk of major bleeding (1.9% of the patients receiving therapeutic-dose anticoagulation and in 0.9% with standard anticoagulation) [20]. Differently, the RAPID trial, which evaluated therapeutic heparin as compared with prophylactic heparin or LMWH in 465 non critically ill patients with elevated D-dimer (approximately twofold the upper limit of reference-ULN) showed no difference between groups in the primary outcome (a composite of ICU admission, noninvasive or invasive mechanical ventilation, or death), although the therapeutic anticoagulation group had a lower incidence of death at 28 days [21]. More recently, The HEP-COVID multicenter randomized clinical trial showed that therapeutic-dose LMWH reduced major thromboembolism and death compared with institutional standard heparin thromboprophylaxis among adult inpatients with COVID-19 and D-dimer levels more than 4 times the upper limit of normal or sepsis-induced coagulopathy score of 4 or greater. This effect was not seen in ICU patients [22]. On the basis of these results, the optimal thromboprophylactic regimen in hospitalized non critically ill patients with COVID-19 with either therapeutic or intermediate heparin dosage remains an open issue [23], as therapeutic doses can be associated with an increased risk of bleeding [19, 20]. On the other hand, in this setting of hospitalized non-critically ill COVID-19 patients, weight adjusted intermediate doses of enoxaparin could reduce thromboembolic complications with improved outcomes and potentially ameliorate the progression of the disease [24]. With this premise, we performed a study aimed to assess the safety of intermediate weight adjusted enoxaparin doses in hospitalized patients with moderate-severe COVID-19 infection. A parallel observational group of patients receiving standard prophylaxis was used to investigate efficacy as secondary aim.

## Methods

### Experimental design

A non-randomized parallel assignment study was conducted with two arms: a phase II single-arm interventional study including all patients treated with the study drug and an observational cohort including all patients screened for receiving the study drug but not included in the phase II study.

### Objectives

The primary objective was to analyze the safety of intermediate weight adjusted enoxaparin in hospitalized patients with moderate-severe COVID-19. Secondary objective was to investigate the efficacy of intermediate weight adjusted enoxaparin in improving the clinical outcome of hospitalized patients with moderate-severe COVID-19.

### Setting

The study INHIXACOVID19 was conducted in 13 Italian centers (see Supplementary Table 1) with the Infectious Disease Unit of the S. Orsola-Malpighi Hospital University of Bologna, a 1.420-bed tertiary care University Hospital in Bologna, as promoting center. The study was approved by the Ethics Committee of the Lazzaro Spallanzani National Institute for Infectious Diseases of Rome which has been attributed the role of National Ethics Committee for assessing clinical trials on medicines for human use and medical devices for patients with COVID-19. The study was approved also by the Italian Drug Agency - AIFA - (EudraCT Number: 2020-001308-40) on 15/04/2020 and was registered on ClinicalTrials.gov with the trial registration number (TRN) NCT04427098 on 11/06/2020. The study was conducted in accordance with the Declaration of Helsinki Ethical Principles and Good Clinical Practices.

### Study population

The same inclusion and exclusion criteria were applied for patients included either in the interventional or in the observational arms. Inclusion criteria were: age >  = 18 y; hospital admission; microbiologically confirmed COVID-19 infection; moderate-severe disease according to study definitions (see below); and ability to provide consent to participate and to use data for interventional study, only to use data for observational cohort. Patients were excluded in case of coagulopathy: INR > 1.5, aPTT ratio > 1.4; impaired renal function (clearance to creatinine less than 15 ml/min); known hypersensitivity to heparin; history of heparin induced thrombocytopenia; presence of an active bleeding or condition susceptible of bleeding in presence of anticoagulation (e.g. recent hemorrhagic stroke, peptic ulcer, malignant tumors at high risk of bleeding, recent neurosurgery or ophthalmic surgery, vascular aneurysms, arteriovenous malformations); body weight < 45 or > 150 kg; concomitant anticoagulant treatment for other indications (e.g. atrial fibrillation, venous thromboembolism, prosthetic heart valves); dual antiplatelet therapy; pregnant or breast-feeding women.

### Clinical severity definitions

Clinical severity of COVID-19 was assessed at the time of diagnosis, during the treatment with the study drug, and at the end of treatment according to the following criteria [25]:

- Mild: only mild symptoms without radiographic features
- Moderate: fever, respiratory symptoms, and radiographic signs of pneumonia
- Severe: fever, respiratory symptoms, and radiographic signs of pneumonia *plus* at least one of three criteria: (1) RR > 30 times/min, (2) oxygen saturation < 93% on ambient air, (3) PaO2/FiO2 < 300 mmHg.
- Critical: meet one of three criteria: (1) respiratory failure needing invasive ventilation, (2) septic shock, (3) multiple organ failure.

### Treatments

Patients included in the observational cohort received standard thromboprophylaxis with enoxaparin 40 mg od, while patients included in the interventional cohort received subcutaneous enoxaparin in a single daily dose of:

- 60 mg (body weight of 45–60 kg)
- 80 mg (body weight of 61–100 kg)
- 100 mg (body weight > 100 kg)

The intermediate dose was chosen to be 1 mg/kg/day which corresponds to half of the recommended enoxaparin therapeutic dose, that is 1 mg/kg twice daily, according to the European Medicines Agency (EMA) technical data sheet of Inhixa [26].

Enoxaparin was to be started on the first day of COVID-19 diagnosis and continued for 14 days, after determination of baseline PT, aPTT, complete blood cell count and creatinine levels. Standard thromboprophylaxis was allowed for a maximum of 72 h before enrollment.

### Laboratory tests

After reaching the steady state (usually after the third dose), heparin levels were measured with the determination of anti-Xa activity on a blood sample obtained at 4 h after the morning injection. LMWH dose could then be increased or reduced according anti-FXa (anti-Xa) activity (0.4–0.6 anti-Xa UI/ml for intermediate doses) [25]. This range was chosen on the basis of previous studies on thromboprophylaxis showing that this is a subtherapeutic range, the therapeutic range of twice daily enoxaparin being 0.6–1.0 UI/ml [27] range which has been shown to be attained with 1 mg/kg/twice daily [28], while the range attained with enoxaparin prohylactic dose is 0.2–04 UI /mL [29].

The determination of anti-Xa activity was repeated on the fifth or sixth day to monitor any drug accumulation. anti-Xa activity was measured according to locally available test. The effective doses of study drugs received by each participant during the study were recorded.

Complete blood cell count was obtained every second day to monitor for heparin induced thrombocytopenia.

D-dimer values were determined locally, and values were expressed as ng/ml in FEU (Fibrinogen Equivalent Units) and those measurements expressed as D-Dimer unit were converted into FEU. D-dimer values were expressed as a ratio of the upper limit of reference value (x ULN). In all patients, RT-PCR nasopharyngeal swabs were performed every 7 days to assess virus clearance and determined locally. All other laboratory tests were determined locally.

### Follow-up

Follow-up was 90 days after study drug initiation. Follow-up information was collected via telephone calls, patient medical records and/or clinical visits according to clinical evolution.

### Outcomes

Primary outcome was the rate of adverse events (AEs) in particular bleeding complications during treatment, at the end of treatment (EOT) and at 30 days after EOT, and occurrence of thromboembolic event at 90 days after COVID-19 diagnosis. Symptomatic thromboembolic events were objectively confirmed deep vein thrombosis (DVT with compression ultrasound), pulmonary embolism (PE with CT pulmonary angiography), stroke (with CT scan), acute myocardial infarction (according to the 4^th^ Universal definition of myocardial infarction), and peripheral acute ischemia. Major bleeding was defined according to the International Society of Thrombosis and Haemostasis (ISTH) criteria as one of the following: fatal bleeding, symptomatic bleeding in a critical area or organ, such as intracranial, intraspinal, intraocular, retroperitoneal, intra-articular or pericardial, or intramuscular with compartment syndrome, bleeding causing a fall in hemoglobin level of 2 g/dL or more or leading to transfusion of two or more units of whole blood or red cells [30]. Secondary outcome was the composite of all-cause-in hospital, 30-day and 90-day mortality rates, evolution of clinical severity during the treatment, ICU admission and length of ICU stay, length of hospital stay, considered as the percentage of patients in the two groups who had a duration of hospitalization of 14 or more days.

### Outcome assessment

The cause of death was evaluated by an independent adjudication safety committee. The evolution of the clinical severity during treatment was assessed by the attending physician. All outcomes related to thromboembolic and bleeding events were assessed by an independent adjudication safety committee with an interim safety analysis after enrolling the first 50 patients.

### Sample size determination

This was a pilot study and an initial sample of 100 patients for the phase II single-arm interventional trial was established.

### Statistical analyses

Binary variables, such as the presence/absence of a given underlying condition, were reported as raw number and prevalence rates along with the *p*-value from Chi-square’s test to compare the prevalence between the interventional and observational cohort. Continuous outcomes were summarized as the median and interquartile range (i.e. 1^st^ and 3^rd^ quartile), or the mean and 95% confidence interval when the distribution of the variable appeared to be normal. Comparisons of continuous variables in the two cohorts were performed using the Wilcoxon-Mann-Whitney test for non-normal variables and the independent sample t-test for normally distributed variables. For efficacy analysis, the outcomes of patients enrolled in the interventional arm were compared with those observed in the observational cohort by propensity score matching (PSm). A propensity score to receive enoxaparin was calculated and two propensity matched groups of patients were obtained. Variables identified for the propensity-score matching were age, gender, BMI, diabetes, COPD, hypertension and disease severity at visit 1 (enrolment). A propensity score matching (PSm) was calculated using the abovementioned variables with third degree polynomials for the variables age and BMI. A 1-to-1 match for each patient in the interventional cohort was identified using nearest neighbour matching with a caliper of 0.5. Differences in 30-day mortality in the two groups were analyzed. The evolution of the clinical severity (progression from moderate to severe, of from severe to critical disease) of COVID-19 between the two groups were further assessed by a competing risk analysis, considering death as competing event. Given the nature of the study, the amount of missing data for each variable of interest were reported. For a given variable, patients with missing data on that variable were excluded from the analysis. Although there were multiple variables of interest and multiple statistical tests were performed, statistical adjustment for type 1 error was not undertaken given the exploratory nature of the analyses at this stage. All analyses were performed using the statistical software SAS v9.4 and significance level of 5%.

## Results

### Patients’ characteristics

**Fig. 1:**
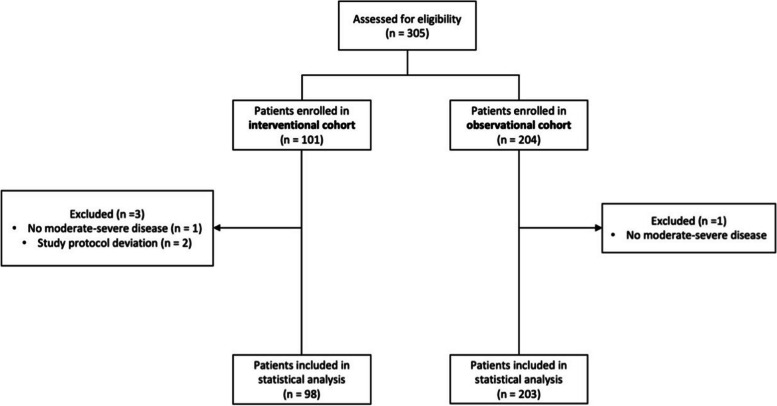
Consort flow diagram illustrating progress of patients throw the study

A total of 305 patients admitted to the hospitals between 08/01/2020 and 12/03/2021 were recruited into the study, 101 in the interventional cohort and 204 in the observational cohort. Contribution on each center is shown in the supplementary Table 1. Among these, 98 patients in the interventional cohort and 203 in the observational cohort were included in the following analyses. One patient in the interventional cohort and another in the observational one were excluded as no moderate-severe disease was diagnosed according to study definitions; two patients in the interventional cohort were further excluded due to study protocol deviations (see Fig. 1). Table 1 shows patients’ characteristics. Patients in the interventional cohort were younger (*p* = 0.001) and more likely to be male (*p* = 0.02) compared to patients in the observational cohort. There were no significant differences regarding ethnicity. Moreover, patients in the interventional cohort had a higher BMI (*p* = 0.005) and Charlson comorbidity index (*p* < 0.001) and were more likely to have hypertension (*p* = 0.003) when compared to patients in the observational cohort. Symptoms and signs were similar among the two groups except for more myalgias, anosmia, ageusia and headache and a lower frequency of PaO_2_/FiO_2_ < 300 among the interventional cohort patients when compared with the observational cohort patients (Table 2). Laboratory parameters, blood pressure, pulse and respiratory rate were similar in the two cohorts (Table 3). D-dimer values were similar in the two groups, and they were only moderately increased (median 682 ng/mL and 1.16 × ULN in 80 subjects in the interventional cohort and 618 ng/mL and 1.32 × ULN in 123 in observational cohort). albeit with missing data. There was no difference in levels of inflammatory markers such as IL-6, fibrinogen, C-Reactive Protein (CRP) between the two groups.

**Table 1:** Characteristics of patients at baseline

	**Interventional cohort**		**Observational cohort**		***P*-value**
	**N**	**Median (IQR) or n (%)**	**N**	**Mean (95% CI) or n (%)**	
**Age [years]**	98	61 (49, 71)	203	66 (56, 77)	< 0.001
**Sex**	98		203		
**Female**		29 (29.6%)		86 (42.4%)	0.03
**Male**		69 (70.4%)		117 (57.6%)	
**Body weight [kg]**	96	80 (74, 90)	163	76 (68, 85)	0.004
**Height [m]**^**a**^	91	1.72 (1.70, 1.74)	150	1.69 (1.68, 1.71)	0.02
**BMI [kg/m**^**2**^**]**^**a**^	91	27.1 (25.3, 30.8)	148	26.3 (24.2, 28.6)	0.005
Charlson index	97	2 (1, 3)	203	3 (1, 5)	< 0.001
**Diabetes mellitus**	98	13 (13.3%)	203	35 (17.2%)	0.41
**COPD**	98	5 (5.1%)	203	23 (11.3%)	0.09
Obesity	98	25 (25.5%)	180	29 (16.1%)	0.08
**Hypertension**	96	36 (37.5%)	203	114 (56.2%)	0.003
Smoking	96	5 (5.2%)	203	9 (4.4%)	0.77
Vaping cigarettes user	5	1 (20.0%)	7	0 (0.0%)	0.42
Alcoholism	96	3 (3.1%)	203	2 (1.0%)	0.33
Active opioid use	96	1 (1.0%)	203	0 (0.0%)	0.32
Haemodialysis	96	0 (0.0%)	203	0 (0.0%)	n/a
Immunocompromised	97	0 (0.0%)	203	7 (3.4%)	0.10

Bold: values used in Propensity Score Matching

^a^Mean and 95% CI are reported and *p*-value was obtained from t-test for independent samples

**Table 2:** Disease symptoms and signs

	**Interventional cohort**		**Observational cohort**		***P*-value**
	**N**	**n (%)**	**N**	**n (%)**	
Fever	98	77 (78.6%)	203	158 (77.8%)	0.99
Respiratory symptoms	98	80 (81.6%)	203	160 (78.8%)	0.65
Radiological evidence of pneumonia	98	97 (99.0%)	203	201 (99.0%)	0.99
Respiratory rate > 30	98	5 (5.1%)	203	7 (3.4%)	0.54
Oxygen saturation < 93%	98	34 (34.7%)	203	72 (35.5%)	0.99
PaO_2_/FiO_2_ < 300	98	33 (33.7%)	203	98 (48.3%)	0.02
Respiratory failure requiring invasive ventilation	98	0 (0.0%)	203	2 (1.0%)	0.99
Septic shock	98	0 (0.0%)	203	0 (0.0%)	n/a
Multiorgan failure	98	0 (0.0%)	203	0 (0.0%)	n/a
Fever > 38.3 °C	98	41 (41.8%)	203	64 (31.5%)	0.09
Cough	98	61 (62.2%)	203	109 (53.7%)	0.17
Dyspnoea	98	43 (43.9%)	203	106 (52.2%)	0.18
Fatigue	98	40 (40.8%)	203	78 (38.4%)	0.71
Myalgia	98	31 (31.6%)	203	25 (12.3%)	< 0.001
Anosmia/Ageusia	98	24 (24.5%)	203	13 (6.4%)	< 0.001
Nausea/Vomiting	98	9 (9.2%)	203	16 (7.9%)	0.82
Diarrhoea	98	18 (18.4%)	203	30 (14.8%)	0.50
Conjunctivitis	98	3 (3.1%)	203	3 (1.5%)	0.40
Confusion	98	4 (4.1%)	203	18 (8.9%)	0.16
Headache	98	17 (17.3%)	203	15 (7.4%)	0.02

**Table 3:** Laboratory tests and vital parameters

	**Interventional cohort**		**Observational cohort**		***P*-value**
	**N**	**Median (IQR) or n (%)**	**N**	**Median (IQR) or n (%)**	
WBC [10^9/mm^3^]	98	6.26 (4.20, 8.51)	202	6.42 (4.50, 8.55)	0.73
RBC [10^12/mm^3^]^a^	98	4.69 (4.58, 4.79)	202	4.58 (4.49, 4.67)	0.17
Hb [g/dL]	98	13.7 (12.7, 14.7)	202	13.5 (12.2, 14.6)	0.26
Neutrophils [10^9/mm^3^]	96	4.53 (2.89, 6.83)	187	4.56 (3.19, 7.11)	0.48
Lymphocytes [10^9/mm^3^]	96	1.10 (0.79, 1.56)	187	0.98 (0.74, 1.47)	0.16
Eosinophils [10^9/mm^3^]	96	0 (0,0)	187	0 (0, 0)	0.40
Platelets [10^9/mm^3^]	97	224 (170, 275)	201	206 (160, 255)	0.08
CRP [mg/dL]	94	8.15 (2.32, 14.50)	196	7.27 (2.66, 12.30)	0.33
PCT [ng/mL]	73	0.1 (0.1, 0.2)	97	0.1 (0.1, 0.2)	0.86
aPTT [ratio]	90	1.02 (0.90, 1.34)	140	1.09 (0.99, 3.64)	0.06
INR	90	1.07 (1.02, 1.12)	181	1.08 (1.02, 1.12)	0.62
**D-dimer [ng/mL]** **x ULN (upper limit of the reference range) mean, (min, max)**	**80**	**682 (355, 963)** **1.16** **2.3 (0.21, 36)**	**123**	**618 (417, 1013)** **1.32** **1.80 (0.32, 12.7)**	**0.28**
Glucose [mg/dL]	94	112 (94, 145)	184	115 (96, 137)	0.95
Urea [mg/dL]	87	35 (27, 46)	136	36 (28, 48)	0.63
Creatinine [mg/dL]	98	0.87 (0.71, 1.02)	200	0.87 (0.72, 1.10)	0.30
AST [IU/L]	95	35 (26, 53)	159	33 (23, 47)	0.14
ALT [IU/L]	97	33 (20, 52)	199	26 (18, 38)	0.06
Bilirubin [mg/dL]	94	0.51 (0.40, 0.76)	142	0.60 (0.42, 0.88)	0.21
LDH [IU/L]	92	281 (233, 358)	169	284 (234, 382)	0.76
Triglycerides [mg/dL]^a^	59	109 (99, 119)	24	128 (109, 148)	0.05
Ferritin [mcg/L]	86	499 (259, 900)	89	448 (217, 753)	0.29
Fibrinogen [mg/dL]	79	546 (471, 633)	80	544 (437, 650)	0.72
CPK [IU/L]	82	97 (58, 189)	99	92 (47, 211)	0.50
Troponin [ng/L]	65	6.0 (5.0, 11.0)	43	8.0 (6.0, 13.0)	0.12
IL-6 [pg/mL]	80	20.0 (7.9, 40.7)	83	25.0 (10.6, 46.6)	0.67
Systolic blood pressure	98	125 (115, 135)	201	125 (120, 140)	0.74
Diastolic Blood Pressure	98	80 (70, 80)	199	75 (70, 80)	0.53
MAP	98	93.3 (84.3, 99.3)	199	93.3 (83.3, 100.0)	0.82
Pulse rate^a^	97	85 (82, 87)	200	83 (82, 85)	0.50
Respiratory Rate	84	20 (18, 24)	145	20 (18, 24)	0.22

^a^Mean and 95% CI are reported and *p*-value was obtained from t-test for independent samples

A total of 66 patients in the interventional cohort (66.0%) received heparin before enrollment in the study for no longer than 48 h; the majority with a dosage of 40 mg (Table 4). Most patients in the interventional cohort (83.0%) received a dosage of 80 mg, while most patients in the observational cohort (97.5%) received a dosage of 40 mg. In 30 subjects (30.6%) of the intervention arm, the heparin dose was adjusted according to the anti-Xa activity, it was decreased in 25, while it was increased in 5 patients but according with the body weight (mean ± SD 100.8 ± 27.6 kg) of patients the dosage remained in the subtherapeutic range. There were 12 patients who were on aspirin, 4 in the interventional group and 8 in the observational group.

**Table 4:** Heparin administration in the interventional and observational cohort

	**Interventional cohort**		**Observational cohort**		***P*-value**
	**N**	**Median (IQR) or n (%)**	**N**	**Median (IQR) or n (%)**	
Pre-enrolment heparin administration	98	66 (66.0%)		n/a	n/a
Dose	64			n/a	n/a
40 mg		45 (70.3%)			
60 mg		10 (15.6%)			
80 mg		8 (12.5%)			
100 mg		1 (1.6%)			
**Heparin dosage**	98		203		< 0.001
40 mg		0 (0.0%)		198 (97.5%)	
60 mg		6 (6.1%)		2 (1.0%)	
**80 mg**		**81 (82.7%)**		**3 (1.5%)**	
100 mg		11 (11.2%)		0 (0.0%)	
Day 3 Anti Xa activity (IU/mL)	69	0.58 (0.48, 0.66)		n/a	n/a
Day 5 Anti Xa activity (IU/mL)	63	0.58 (0.47, 0.65)		n/a	n/a
**Heparin new dosage [mg]**	**30**			**n/a**	**n/a**
40 mg		3 (10.0%)			
60 mg		19 (63.3%)			
80 mg		4 (13.3%)			
100 mg		4 (13.3%)			

Regarding other treatments for COVID-19, a similar proportion of subjects received steroids, remdesivir and tocilizumab in the interventional and observational arms (81 vs. 78%, 37 vs. 27% and 8 vs. 12% respectively).

### Analysis of safety

The bleeding events within 30 days were 5.4% and 0.5% in the interventional and in the observational arms (*p* = 0.02), respectively (see Table 5). However, only one out of the five events observed in the interventional arm was major (melena) and occurred in a patient also on aspirin, while all the remaining bleedings were adjudicated as non-major (3 epistaxis and one rectal bleed in the interventional cohort and 1 epistaxis in the observational cohort). At 90 days 2.2% and 1.6% (*p* = 1) of patients in the interventional and observational cohort, respectively, had bleeding; these were all non-major bleedings. All non-major bleedings were minimal and self-limiting.

**Table 5:** Bleeding and thromboembolic events in the interventional and observational cohorts

	**Interventional cohort**		**Observational cohort**		***P*-value**
	**N**	**n (%)**	**N**	**n (%)**	
Bleeding at 30 days	93	5 (5.4%)	186	1 (0.5%)	0.02
		Minor 4 (80%)		Minor 1 (100%)	
		Major 1 (20%)		Major 0 (0%)	0.33
Thromboembolic events at 30 days	92	0 (0.0%)	186	4 (2.2%)	0.31
Bleeding at 90 days	93	2 (2.2%)	184	3 (1.6%)	1.00
Thromboembolic events at 90 days	93	3 (3.2%)	184	0 (0.0%)	0.04

At 30 days, four patients in the observational cohort had a thromboembolic event; of these two were venous thrombosis and two were pulmonary embolism. At 90 days, there were 3 thromboembolic events among patients in the interventional cohort; one patient had both venous thrombosis and pulmonary embolism, two patients had venous thrombosis only. Patients were not routinely evaluated for thromboembolic events in the observational cohort at 90 days.

### Analysis of efficacy

Crude comparison of outcome variables between interventional and observational arms are shown in supplementary Table 2. The rate of clinical improvement across the study visits was higher in the interventional group than in the observational cohort (65.3 vs. 52.2%, *p* = 0.03). There were no difference in all-cause mortality at 30 and 90 days; rates of ICU admission were 13.3% and 6.9% (*p* = 0.09) in interventional and observational arms, respectively; length of hospital stay was shorter in the interventional arm [13(8–16) vs. 14 (11–22), *p* < 0.001] (see supplementary Fig. 1 in supplementary material).

To account for differences in the two groups, a propensity score matching algorithm was done identifying a match for 90 patients both in the interventional and observational cohort, based on age, gender, BMI, diabetes, COPD, hypertension and disease severity at enrollment. Baseline characteristics in the matched groups showed no statistical differences between the two groups after matching (see supplementary Table 3 in supplementary material).

**Fig. 2:**
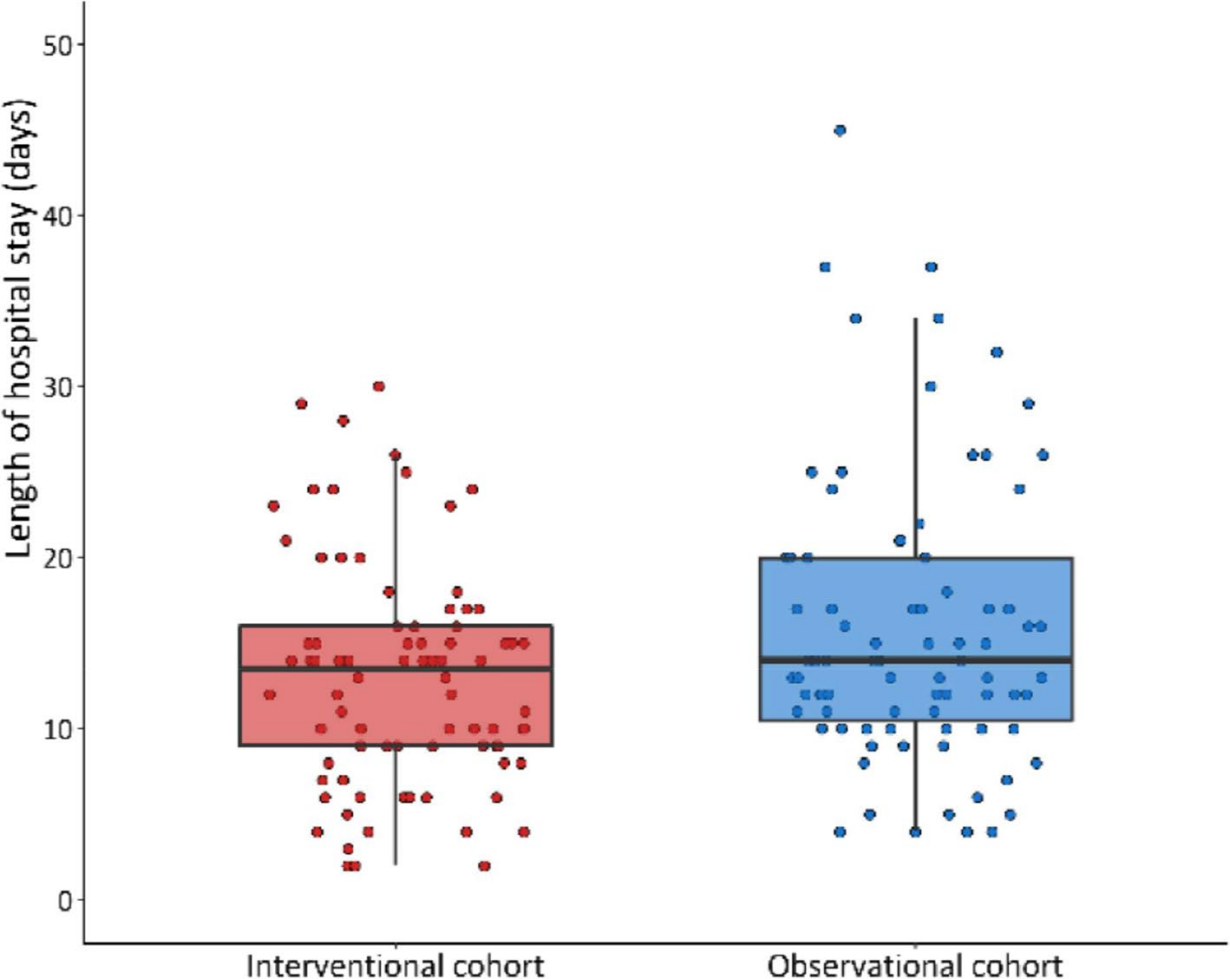
Length of hospital days in patients in the interventional and observation cohort after propensity-score matching

Mortality at 30 and 90 days did not differ significantly between the two cohorts (*p* = 0.37 at both 30 and 90 days, Table 6). Rates of ICU admission were 13.3% in the interventional cohort and 8.9% in the observational cohort (*p* = 0.48). Improvement of clinical status between enrollment and day 14 was observed in 64% of patients in the interventional cohort and 57.8% in the observational cohort (*p* = 0.36). A trend toward lower length of stay in the interventional cohort compared to observational cohort was observed (*p* = 0.08) (Fig. 2).

**Table 6:** Mortality, ICU admission and related events after propensity score matching

	**Interventional cohort**		**Observational cohort**		***P*-value**
	**N**	**Median (IQR) or n (%)**	**N**	**Median (95% CI) or n (%)**	
Death within 30 days from heparin onset	88	3 (3.4%)	90	1 (1.1%)	0.37
Cause of death at 30 days	3		1		
COVID-19		2 (66.7%)		1 (100.0%)	
COVID-19 complications		1 (33.3%)		0 (0.0%)	
Death within 90 days from heparin onset	89	3 (3.4%)	90	1 (1.1%)	0.37
Cause of death at 90 days	3		1		
COVID-19		2 (66.7%)		1 (100.0%)	
COVID-19 complications		1 (33.3%)		0 (0.0%)	
Length of hospitalization [days]	85	14 (9, 16)	85	14 (10, 20)	0.08
NIV	90	14 (15.6%)	90	10 (11.1%)	0.51
CPAP	90	11 (12.2%)	90	11 (12.2%)	1.00
High nasal O_2_ flow	90	16 (17.8%)	90	14 (15.6%)	0.84
ICU admission	90	12 (13.3%)	90	8 (8.9%)	0.48
Length of ICU stay [days]	8	8 (5, 17)	8	6 (5, 10)	0.76
Death within 90 days or ICU admission	89	13 (14.6%)	90	9 (10.0%)	0.37
Improvement of clinical status between visit 1 and visit 3	90	58 (64.4%)	90	52 (57.8%)	0.36

The composite outcome of all-cause mortality at 30-day and 90-day, evolution of the clinical severity during treatment, ICU admission, length of hospital stay was calculated. The composite outcome was 54% (53 /98) in the interventional cohort and 65% (132/203) in the observational cohort (*p* = 0.07) before PSm, while it was 55.6% (50/90) in the interventional cohort and 62.2% (56/90) (*p* = 0.45) after PSm (Table 6).

## Discussion

This phase II pilot study showed that thromboprophylaxis with intermediate dose heparin with anti-FXa monitoring in patients with moderate or severe COVID-19 and moderately increased D-dimer was not associated with a higher risk of major bleeding compared to patients receiving standard dose prophylaxis. Indeed, there was only one major bleeding event in the interventional cohort in a subject on aspirin. Our results suggest that intermediate dose heparin with anti-FXa adjustment could be considered safe. Regarding efficacy assessment, although at the unadjusted analysis the rates of clinical improvement over study visits and the length of hospital stay were more favorable in the interventional group patients, their significance was reduced after the propensity score matching.

Differently from the RAPID [21] and HEP-COVID [22] studies in which non critically ill patients with high D-dimer were enrolled (D-dimer 2.3 × ULN in the RAPID and D-dimer levels more than 4 times ULN in the HEP-COVID study), in this cohort patients were enrolled with only moderately increased D-dimer levels (median 1.1–1.3 × ULN). This indicates that patients at lower risk were enrolled as reflected by low mortality rates at 30 days (3% in the interventional cohort and 1% in the observational cohort). Mortality rates were higher both in the RAPID trial (7.6% in the standard prophylactic dose) and the HEP-COVID (19.4%) trial. In the ATTACC, ACTIV-4a, and REMAP-CAP multiplatform trial in non-critically ill patients hospitalized with COVID-19, therapeutic heparin increased the probability of survival to hospital discharge with reduced use of cardiovascular or respiratory organ support as compared with usual-care thromboprophylaxis [19, 20]. However, in this trial 77% of subjects had high D-dimer levels with 7–8% in- hospital mortality indicating a higher risk patient group. In this trial therapeutic dose heparin was also associated with a higher risk of major bleeding (1.9%) when compared with standard prophylaxis.

Several other randomized clinical trials have been conducted evaluating increased subtherapeutic doses or intermediate dose of LMWH in comparison with standard dose prophylaxis such as the study by Perepu et al. [31] and the X–COVID 19 [32]. These studies however had all reduced sample size and were underpowered to show an effect of different doses of LMWH on mortality or thromboembolic events. The BEMICOP trial compared therapeutic dose with standard prophylactic dose bemiparin in only 65 patients with nonsevere COVID-19 pneumonia and elevated D-dimer. without showing any difference of clinical outcomes between the two regimens [33].

Several systematic reviews and meta-analyses have also been conducted to assess the efficacy and safety of intermediate/therapeutic in comparison with standard prophylactic doses in hospitalized patients with COVID-19 [34–38].

Five-thousand four-houndred and five hospitalized patients with COVID-19 were analyzed in a meta-analysis including 8 randomized controlled trials. This meta-analysis showed a statistically significant reduction in the odds of development of thrombotic events in patients underwent to intermediate dose prophilaxis or to therapeutic anti-coagulation relative to ones underwent to prophylactic anticoagulation. However, the main problem was an increased risk of bleeding [34]. A subgroup analysis of randomized trials which recruited only patients with severe SARS-CoV-2 infection treated with intermediate or therapeutic dose of anticoagulation, has demonstrated a statistically significant reduction in the odds of development of thrombotic events with no-effect on mortality and an increased risk of bleeding [34]. More recently, 11.430 hospitalized patients were included in a meta-analysis of 6 randomized controlled trials and 25 cohort studies. This meta-analysis showed that risk for all-cause mortality was higher when standard prophylaxis was applied and lower when patients received an intermediate-dose LMWH prophylaxis. However, no associations were detected between the intensity of LMWH and the risk of thrombotic and hemorrhagic events, except the lower risk for hemorrhage in patients on prophylactic compared to higher LMWH doses [35].

A meta-analysis including 33 studies (31 observational, 2 RCT) for a total overall population of 32,688 patients showed that both prophylactic and full dose heparins reduced mortality. However, the full dose was associated with a higher risk of major bleeding compared to prophylactic dose [36].

A Cochrane systematic review included seven RCT (16,185 participants) and showed little to no difference in all-cause mortality or DVT with higher dose UFH, LMWH or rivaroxaban when compared with standard thromboprophylaxis with higher risk of minor bleeding and a slightly higher risk of major bleeding [37].

Finally, a systematic review including a total of 5470 patients from 9 RCTs showed that in hospitalized patients with COVID-19, high-dose thromboprophylaxis with either UFH or LMWH or fondaparinux is more effective than low-dose for the prevention of VTE but increases the risk of major bleeding [38].

The most important results of the INHIXACOVID study appear to be the safety of intermediate dose heparin. anti-Xa adjustment led to a change of dosage in 30 patients and this seems to have increased the safety of intermediate dose heparin. No serious adverse event was correlated to the study drug.

Regarding a potential favorable impact on COVID-19 course, although the length of hospitalization was lower in the interventional cohort compared to the observational cohort (median 13 vs. 14 days, *p* < 0.001), after PS matching this difference was not statistically significant. This finding appears interesting considering the lack of such observation in published studies in critically ill patients and / or in ICUs.

The main limitation of this study is the lack of a randomized control group. However, at the time of trial design, a double blind randomized clinical trial was deemed not feasible, to limit the burden on extremely strained healthcare systems because of the pandemic, especially in Italy. Another limitation is the limited sample size with reduced statistical power and the lack of assessment of thromboembolic complications at 90 days of the patients of the observational cohort. Patient’s characteristics may have influenced the decision to enroll patients (selection bias). In addition, clinicians’ decisions to admit patients to ICU or treat them with non-invasive mechanical ventilation could have been influenced by knowledge of the allocated treatment (performance bias). Moreover, random variation could have been caused by the pandemic’s variable impact on hospital resources over time and across regions. Investigation or reporting of potential events (especially incidental deep vein thromboses or bleeding) in patients receiving intermediate heparin could have been less likely than events in patients receiving prophylactic heparin (detection bias). However, an independent clinical events committee blindly adjudicated all relevant outcomes based on clinical reports. Propensity score matching was employed to reduce or eliminate the effects of confounding when using observational data to estimate treatment effects and it was based on those variables which were different at baseline in the two cohorts.

## Conclusions

Intermediate dose heparin with anti-Factor Xa monitoring in patients with moderate or severe COVID-19 and moderately increased D-dimer was not associated with an increased risk of major bleeding. Our data seems to suggest a more favorable clinical course and shorter length of hospitalization in patients treated with intermediate dose heparin as revealed by other studies in the literature.

### Supplementary Information

**Additional file 1: Supplementary Table 1.** Participating centers and relative contribution.

**Additional file 2: Supplementary Table 2.** Crude comparison of outcome variables between interventional and observational groups.

**Additional file 3:** Length of hospital days in patients in the interventional and observation cohort before propensity score matching.

**Additional file 4: Supplementary Table 3.** Baseline characteristics in the propensity score matching groups.

## Data Availability

The datasets generated and/or analysed during the current study are not publicly available due to local privacy policy but are available from the corresponding author on reasonable request.

## Abbreviations

COVID-19: Coronavirus Disease 19
VTE: Venous thromboembolism
ICUs: Intensive care units
LMWH: Low molecular weight heparin
UFH: Unfractionated heparin
EMA: European Medical Agency
FEU: Fibrinogen equivalent units
AEs: Adverse events
EOT: End of treatment
DVT: Deep vein thrombosis
PE: Pulmonary embolism
ISTH: International Society of Thrombosis and Haemostasis
PSm: Propensity score matching
FXa: Factor Xa
