# Supplementary material for: Intermediate dose enoxaparin in hospitalized patients with moderate-severe COVID-19: a pilot phase II single-arm study, INHIXACOVID19
Source: BMC Infect Dis. 2023 Oct 24;23:718. doi: 10.1186/s12879-023-08297-7 (PMC10594805; doi:10.1186/s12879-023-08297-7)
Supplement: Supplementary file 1 — Additional file 1: Supplementary Table 1. Participating centers and relative contribution. [file 12879_2023_8297_MOESM1_ESM.docx]

**Supplementary Table 1: participating centers and relative contribution.**

| **Centre** | **Interventional cohort**  **(n=98)** | **Observational cohort**  **(n=203)** | **Total**  **(n=303)** |
| --- | --- | --- | --- |
| Bologna | 44 (44.9%) | 71 (35.0%) | 115 (38.2%) |
| Brescia | 3 (3.1%) | 8 (3.9%) | 11 (3.7%) |
| Catania | 0 (0.0%) | 17 (8.4%) | 17 (5.6%) |
| Cremona | 5 (5.1%) | 12 (5.9%) | 17 (5.6%) |
| Mantova | 4 (4.1%) | 11 (5.4%) | 15 (5.0%) |
| Opera di San Pio da Pietralcina | 3 (3.1%) | 4 (2.0%) | 7 (2.3%) |
| Opera Amedeo di Savoia | 4 (4.1%) | 0 (0.0%) | 4 (1.3%) |
| Parma | 5 (5.1%) | 9 (4.4%) | 14 (4.6%) |
| Poliambulanza | 2 (2.0%) | 2 (1.0%) | 4 (1.3%) |
| Potenza | 0 (0.0%) | 9 (4.4%) | 9 (3.0%) |
| Rimini Forlí Cesena | 3 (3.1%) | 33 (16.3%) | 36 (12.0%) |
| San Raffaele di Milano | 11 (11.2%) | 21 (10.3%) | 32 (10.6%) |
| Verona | (14.3%) | 6 (3.0%) | 20 (6.6%) |
