# Supplementary material for: Intermediate dose enoxaparin in hospitalized patients with moderate-severe COVID-19: a pilot phase II single-arm study, INHIXACOVID19
Source: BMC Infect Dis. 2023 Oct 24;23:718. doi: 10.1186/s12879-023-08297-7 (PMC10594805; doi:10.1186/s12879-023-08297-7)
Supplement: Supplementary file 2 — Additional file 2: Supplementary Table 2. Crude comparison of outcome variables between interventional and observational groups. [file 12879_2023_8297_MOESM2_ESM.docx]

**Supplementary Table 2. Crude comparison of outcome variables between interventional and observational groups.**

|  | **Interventional cohort** | | **Observational cohort** | | **p-value** |
| --- | --- | --- | --- | --- | --- |
|  | **N** | **Median (IQR)**  **or n (%)** | **N** | **Median (IQR)**  **or n (%)** |  |
| **Death within 30 days from heparin onset** | **96** | **3 (3.1%)** | **200** | **4 (2.0%)** | **0.69** |
| Cause of death at 30 days | 3 |  | 4 |  |  |
| COVID-19 |  | 2 (66.7%) |  | 3 (75.0%) |  |
| COVID-19 complications |  | 1 (33.3%) |  | 1 (25.0%) |  |
| **Death within 90 days from heparin onset** | **97** | **3 (3.1%)** | **202** | **7 (3.5%)** | **1.00** |
| Cause of death at 30 days | 3 |  | 7 |  |  |
| COVID-19 |  | 2 (66.7%) |  | 3 (42.9%) |  |
| COVID-19 complications |  | 1 (33.3%) |  | 3 (42.9%) |  |
| Other |  | 0 (0.0%) |  | 1 (14.3%) |  |
| **Length of hospitalization [days]** | **93** | **13 (8, 16)** | **190** | **14 (11, 22)** | **<0.001** |
| NIV | 98 | 15 (15.3%) | 203 | 22 (10.8%) | 0.27 |
| CPAP | 98 | 11 (11.2%) | 203 | 31 (15.3%) | 0.38 |
| High nasal O_2_ flow | 98 | 18 (18.4%) | 203 | 35 (17.2%) | 0.87 |
| ICU admission | 98 | 13 (13.3%) | 203 | 14 (6.9%) | 0.09 |
| Length of ICU stay [days] | 9 | 9 (5, 21) | 14 | 7 (6, 7) | 0.51 |
| Death within 90 days or ICU admission | 97 | 14 (14.4%) | 202 | 21 (10.4%) | 0.34 |
| Mechanical ventilation | 12 | 5 (41.7%) | 14 | 4 (28.6%) | 0.68 |
| ECMO | 11 | 7 (63.6%) | 13 | 6 (46.2%) | 0.44 |
| CVVH | 11 | 0 (0.0%) | 13 | 0 (0.0%) | n/a |
