# Supplementary figures and images for: Intermediate dose enoxaparin in hospitalized patients with moderate-severe COVID-19: a pilot phase II single-arm study, INHIXACOVID19
Source: BMC Infect Dis. 2023 Oct 24;23:718. doi: 10.1186/s12879-023-08297-7 (PMC10594805; doi:10.1186/s12879-023-08297-7)

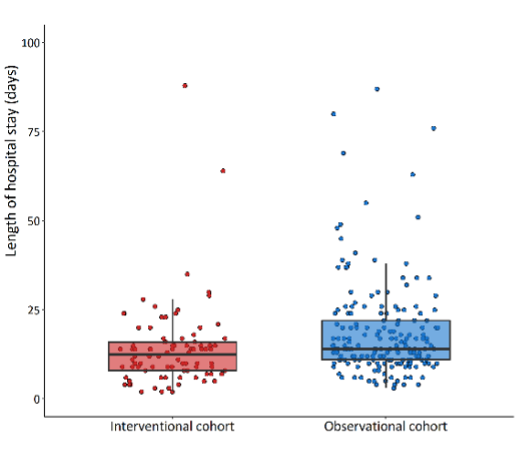

Supplement: Supplementary file 3 — Additional file 3: Length of hospital days in patients in the interventional and observation cohort before propensity score matching. [file 12879_2023_8297_MOESM3_ESM.png]
