# Supplementary material for: Intermediate dose enoxaparin in hospitalized patients with moderate-severe COVID-19: a pilot phase II single-arm study, INHIXACOVID19
Source: BMC Infect Dis. 2023 Oct 24;23:718. doi: 10.1186/s12879-023-08297-7 (PMC10594805; doi:10.1186/s12879-023-08297-7)
Supplement: Supplementary file 4 — Additional file 4: Supplementary Table 3. Baseline characteristics in the propensity score matching groups. [file 12879_2023_8297_MOESM4_ESM.docx]

**Supplementary Table 3. Baseline characteristics in the propensity score matching groups.**

|  | **Interventional cohort** | | **Observational cohort** | | **p-value** | **SMD or**  **RR** |
| --- | --- | --- | --- | --- | --- | --- |
|  | **N** | **Median (IQR)**  **or n (%)** | **N** | **Median (IQR)**  **or n (%)** |  |  |
| Age [years] | 90 | 60 (57, 63) | 90 | 60 (57, 63) | 0.81 | 0.04 |
| Sex  Female  Male | 90 | 25 (27.8%)  65 (72.2%) | 90 | 26 (28.9%)  64 (71.1%) | 0.87 | 1.01 |
| BMI [Kg/m^2^] | 90 | 27.1 (25.3, 29.0) | 90 | 27.3 (25.4, 28.5) | 0.54 | -0.12 |
| Diabetes mellitus | 90 | 13 (14.4%) | 90 | 19 (21.1%) | 0.33 | 0.68 |
| COPD | 90 | 5 (5.6%) | 90 | 6 (6.7%) | 1.00 | 0.84 |
| Obesity | 90 | 20 (22.2%) | 86 | 17 (19.8%) | 0.72 | 1.12 |
| Hypertension | 90 | 33 (36.7%) | 90 | 36 (40.0%) | 0.76 | 0.92 |
| Smoking | 88 | 5 (5.7%) | 90 | 3 (3.3%) | 0.49 | 1.72 |
| Alcoholism | 88 | 3 (3.4%) | 90 | 2 (2.2%) | 0.68 | 1.55 |
| Active opioid use | 88 | 1 (1.1%) | 90 | 0 (0%) | 0.49 | n/a |
| Haemodialysis | 88 | 0 (0%) | 90 | 0 (0%) | n/a | n/a |
| Immunocompromised | 89 | 0 (0%) | 90 | 2 (2.2%) | 0.5 | n/a |
| D-dimer [ng/mL] | 90 | 766 (380, 885) | 90 | 885 (530, 885) | 0.25 | 0.00 |
| Severity at visit 1  Mild  Moderate  Severe | 90 | 1 (1.1%)  58 (64.4%)  31 (34.4%) | 90 | 1 (1.1%)  56 (62.2%)  33 (36.7%) | 0.95 |  |
